# The origin of exotic pet sugar gliders (*Petaurus breviceps*) kept in the United States of America

**DOI:** 10.7717/peerj.6180

**Published:** 2019-01-08

**Authors:** Catriona D. Campbell, Jill Pecon-Slattery, Rebecca Pollak, Leo Joseph, Clare E. Holleley

**Affiliations:** 1 Australian National Wildlife Collection, CSIRO National Research Collections Australia, Canberra, Australian Capital Territory, Australia; 2 Laboratory of Genomic Diversity, National Cancer Institute—National Institutes of Health, Frederick, MD, United States of America; 3 Smithsonian Conservation Biology Institute—National Zoological Park, Front Royal, VA, United States of America; 4 Institute for Applied Ecology, University of Canberra, Bruce, Australian Capital Territory, Australia

**Keywords:** Exotic pets, Sugar glider, Marsupials, Introduced species, *Petaurus breviceps*, Wildlife trade, Importation, Exportation, Mammals

## Abstract

The demand for exotic non-domesticated animals kept as pets in the United States of America (USA) is increasing the exportation rates of these species from their native ranges. Often, illegal harvesting of these species is used to boost captive-bred numbers and meet this demand. One such species, the sugar glider (*Petaurus breviceps*), endemic to Australia and New Guinea is a popular domestic pet due to its small size and “cute” demeanour. Despite a legal avenue for trade existing in Indonesia, concerns have been raised that sugar gliders may be entering the USA from other parts of their native range where exportation is prohibited such as Australia, Papua New Guinea and the surrounding Indonesian islands. We compared previously published DNA sequences from across the native range of sugar gliders with samples collected from domestically kept sugar gliders within the USA to determine provenance and gene flow between source and introduced populations. Here we show that as predicted, the USA sugar glider population originates from West Papua, Indonesia with no illegal harvesting from other native areas such as Papua New Guinea or Australia evident in the samples tested within this study.

## Introduction

The importation of wild animals into the United States of America (USA) has been occurring for many decades. This arises from the popularity and desire for unusual specimens, traditional medicines, the entertainment industry and companion animals (Nekaris et al., 2010; Nijman, 2010; Bush, Baker & Macdonald, 2014). In the USA it is common for non-domesticated animals to be kept as small companion pets, examples include North American black-tailed prairie dogs (*Cynomys ludovicianus*), African pygmy hedgehogs (*Atelerix albiventris*), reptiles and the focus of this manuscript, sugar gliders (*Petaurus breviceps*) (Johnson-Delaney, 2006). It is generally assumed that commercial pet breeders within the USA stock animals through legal channels, however illegal animal exportation continues to occur from both developed and developing countries (Alacs & Georges, 2008; Wyler & Sheikh, 2008). A common source of illegal wildlife trade in the pet market is the false classification of illegally harvested wild-caught animals as ‘farmed’ or ‘captive-bred’ animals (Bulte & Damania, 2005; Mockrin, Bennett & Labruna, 2005). This means that despite adhering to legal importation channels, USA pet breeders could unwittingly be supporting illegal wildlife trade. Illegal wildlife trade is a significant ethical and economic issue and is an industry worth between $5 billion and $23 billion US dollars per year (Wyler & Sheikh, 2008; World Economic Forum, 2017).

The sugar glider (*Petaurus breviceps*) is a small arboreal and nocturnal marsupial whose native distribution includes continental Australia and the island of New Guinea (Smith, 1973; Malekian et al., 2010) as well as an introduced population in Tasmania, Australia (Campbell et al., 2018). Currently, seven morphologically defined subspecies are recognized across the species’ range. Four occur on the island of New Guinea (*P. b. flavidus, P. b. papuanus, P. b. taxa, P. b. biacensis*) and three are in Australia (*P. b. ariel*, *P. b. longicaudatus, P. b. breviceps*). This taxonomy, however, is not supported by a previous mitochondrial study (Malekian et al., 2010). Sugar gliders usually nest in social groups of between two and seven but are known to nest alone on occasion (Suckling, 1984). They occur in rainforests and wet and dry sclerophyll forests and are hollow-dependent (Koch, Munks & Woehler, 2008). Home range varies between seasons and fluctuates between 0.3 and 2.8 hectares (Suckling, 1984). Diet consists of the sap of *Eucalyptus spp*., *Acacia spp.* gum, leaves of host trees, invertebrates and honeydew produced by insects (Smith, 1982; Suckling, 1984). An introduced population of sugar gliders on Australia’s island state of Tasmania also has carnivorous dietary preferences, including hollow-nesting birds, their eggs and young (Stojanovic et al., 2014).

The sugar glider was brought to the USA for the exotic pet trade in the 1990s (Brust, 2009). The current size of the USA sugar glider population is unknown, but it makes up a proportion of the 3.5 million exotic mammalian pets kept in private households (excluding ferrets, rabbits and livestock) (American Veterinary Medical Association, 2018). This established USA population may have been founded by a small number of individuals and possibly from only a small part of the native range. The prevailing view from active breeders and sugar glider enthusiast websites is that the USA population of sugar gliders originates from West Papua, Indonesia, on the island of New Guinea (Table S1). There is, however, little to no documentation to substantiate this anecdotal view. Additionally, there is the possibility that Australian sugar gliders were imported into the USA before exportation was banned in 1982 with the implementation of the Wildlife Protection Act (Regulation of Exports and Imports) 1982, or that undocumented or illegal trade of Australian populations may have occurred and/or be on going (C Johnson-Delaney, pers. comm., 2010). Thus, the provenance of the USA population of sugar gliders is essentially unknown and no data are available on its genetic diversity.

Sugar gliders can be legally traded internationally from breeding facilities based in Jakarta, which are known to supply the pet trades in Malaysia, Thailand and USA (Lyons & Natusch, 2012). These animals are thought to be wild trapped from the Sorong area (West Papua, Indonesia) as part of a quota system but there is no documentation about where individual animals were trapped (Lyons & Natusch, 2011; Lyons & Natusch, 2012). In this study we use nuclear and mitochondrial markers to assess the provenance and genetic diversity of sugar gliders imported to the USA. We examine three possible origins for historical and ongoing importation: (a) sugar gliders originate from West Papua, Indonesia and are thus legally imported; (b) sugar gliders are traded from West Papua into the USA but are wild-caught elsewhere in Indonesia or Papua New Guinea and; (c) sugar gliders are wild-caught in Australia and smuggled into the USA, either directly or through an intermediate hub. We characterise the genetic diversity residing within the USA population and compare it to other independent introduction events.

## Materials and Methods

### Sample collection

**Figure 1:**
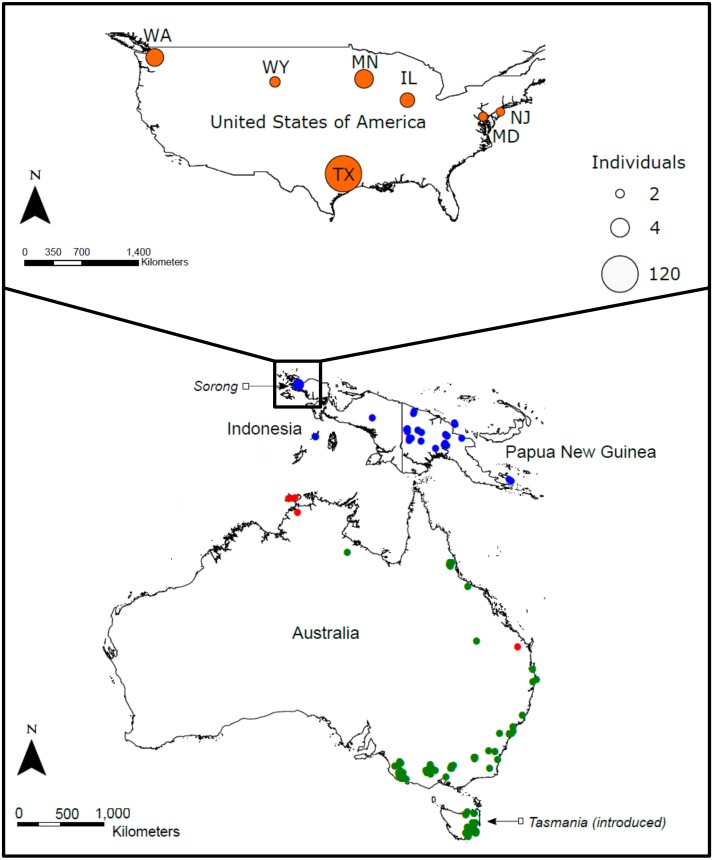
*Petaurus breviceps* samples collected from the pet trade in the United States of America and wild-caught specimens from across the species native range. The introduced USA population is indicated in orange, and native populations on the Island of New Guinea in blue (*P. b papuanus, P. b. tafa, P. b. flavidus, P. b. biacensis*), Australia in red (*P. b ariel*) and green (*P. b. longicaudatus*, *P. b. breviceps*) (Malekian et al., 2010; Campbell et al., 2018).”

Within the USA, samples were opportunistically collected from the tissue by-product of neuter procedures, thus all samples were from male individuals. Samples were collected from seven states: Washington (*n* = 4), Illinois (*n* = 3), Maryland (*n* = 2), Minnesota (*n* = 4), New Jersey (*n* = 2), Wyoming (*n* = 2) and Texas (*n* = 128) (Fig. 1; Table S2). All tissues were donated by qualified veterinarians, with the identity of the owners, breeders or operators remaining anonymous. The sampling co-ordinates in Table S2 refer to the site of tissue collection. Extensive sampling was possible in Texas with the co-operation of two large commercial operators of sugar glider farms. These breeding facilities are thought to be major suppliers of pet sugar gliders for the greater USA.

To identify the provenance of the USA population, we compiled a database of 93 previously published geo-referenced mitochondrial (mtDNA) and nuclear DNA (nDNA) sequences for *P. breviceps* from Genbank (Benson et al., 2013) in Geneious 10.0.5 (Kearse et al., 2012)*.* Sampling across the native range was comprehensive and included one small introduced population, located on the island state of Tasmania, Australia. Sample sizes for the following locations were as follows: mainland Australia, *n* = 41; Tasmania, *n* = 21; Indonesia, *n* = 5 ; Papua New Guinea, *n* = 26) (Malekian et al., 2010; Campbell et al., 2018) (Fig. 1; Table S2).

### DNA extraction and PCR amplification

DNA was extracted from 145 tissue samples (testes) using the Qiagen Puregene^®^ Tissue Kit following the manufacturer’s protocols. A total of 2,092 bp of DNA were sequenced from two mitochondrial genes (ND2, ND4, 1,394 bp) and one nuclear gene (*ω*-globin, 698 bp) and were amplified to complement previous phylogenetic studies of the sugar glider (Malekian et al., 2010; Campbell et al., 2018). A 695 bp fragment of the mtND2 gene was targeted using primers mmND2.1 (5′-GCACCATTCCACTTYTGAGT-3′) and mrND2c (5′-GATTTGCGTTCGAATGTA-GCAAG-3′) (Osborne & Christidis, 2001). An 699 bp fragment of the mtND4 gene was targeted using primers mt10812H (5′-TGACTACCAAAAGCTCATGTAGAAGC-3′) and mt11769L (5′-TTTTACTTGGATTTGCACCA-3′) (Arevalo, Davis & Sites, 1994) and a 698 bp fragment of the nuclear *ω*-globin gene was targeted using primers G314 (5′-GGAATCATGGCAAGAAGGTG-3′) and G424 (5′-CCGGAGGTGTTYAGTGGTA- TTTTC-3′) (Wheeler et al., 2001). PCR amplifications contained 50 ng of DNA, 1×PCR buffer; 62.5 mM MgCl_2_; 5mM dNTP’s; 0.4 µM each forward and reverse primer; 4 mg BSA, 0.5 µl of Amplitaq Gold and ddH_2_0 to a total volume of 25 µl. PCR conditions consisted of denaturation at 95 °C for 9 min followed by 35 cycles of denaturation at 94 °C for 45 s, 50 °C for 45 s and 72 °C for 45 s and finally an extension period of 72 °C for 10 min. To confirm amplification, 5 µl of PCR product was visualised in a 1.0% agarose gel containing ethidium bromide.

### Sequencing

PCR products were purified using ExoSAP protocol consisting of 20 µl of PCR product combined with 0.72 µl shrimp alkaline phosphatase (SAP); 0.36 µl exonuclease I (EXO); 3.92 µl ddH_2_O (Amersham Pharmacia, Piscataway, NJ, USA), incubated at 37 °C for 30 mins followed by an enzyme inactivation step at 80 °C for 15 mins. Sequencing reactions consisted of 1 µl of purified PCR product, 0.25 µl of BigDye^®^ v3.1 (Applied Biosystems), 1×  sequencing buffer, 0.16 µM primer and ddH_2_0 to a total volume of 9 µl. Cycling conditions were 45 cycles of 96 °C for 10 s, 50 °C for 5 s followed by 60 °C for 4 min. Sequencing reactions were purified using the Agencourt^®^ CleanSEQ dye-terminator removal method as per the manufacturer’s guidelines (Beckman Coulter, Brea, CA, USA). Sequencing was performed on an ABI 3730 DNA Analyser (Applied Biosystems, Foster City, CA, USA) at the National Cancer Institute-Frederick.

### Phylogenetic analysis

Forward and reverse raw sequences were checked and edited manually in Sequencher v4.8 (GeneCodes). The consensus sequences for all individuals were aligned in MUSCLE (Edgar, 2004) using Geneious 10.0.5 (Kearse et al., 2012). Based on the work of Malekian et al. (2010), *Petaurus abidi* was used as the outgroup as it is the closest sister group to *P. breviceps*.

Mitochondrial data and nuclear data were separated for the network analysis because they have different modes of inheritance. Networks were generated using median joining network analysis (Bandelt, Forster & Röhl, 1999) with software package PopART (Allan Wilson Centre Imaging Evolution Initiative) on the concatenated mitochondrial genes ND2 and ND4 and the nuclear *ω*-globin. Number of haplotypes, number of variable sites, haplotype diversity, nucleotide diversity, Tajima’s D (Tajima, 1989) and gene flow indices *F*_ST,_ D_XY,_ and D_A_ for the mitochondrial data were calculated in DnaSP v5.10.1 (Librado & Rozas, 2009).

Phylogenetic analyses were estimated using the maximum likelihood method for the concatenated dataset. Appropriate models of DNA substitution were determined in MEGA v7.0 (Kumar, Stecher & Tamura, 2016) with genes partitioned, using the Akaike Information Criterion (AIC) with gamma distribution and the proportion of invariant sites estimated during the search (Table S3). Maximum likelihood phylogenies were carried out using Garli v2.01 (Zwickl, 2006), which allows for the analysis of partitioned data. Bootstrap support was calculated in Garli v2.01 by performing 1,000 bootstrap replicates. Split support was calculated using DendroPy (Sukumaran & Holder, 2010) and sumtrees v4.0.0 (Sukumaran & Holder, 2015).

## Results

**Figure 2:**
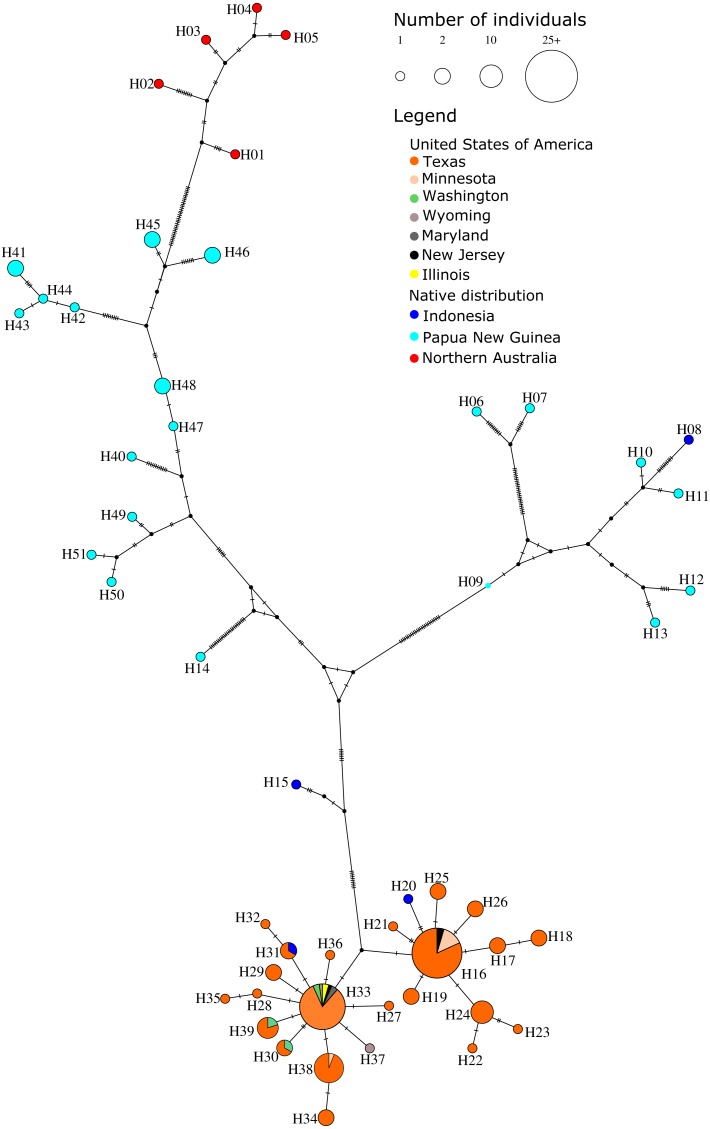
Median joining mitochondrial network demonstrating that *Petaurus breviceps* samples collected from the pet trade in the United States of America are most closely related to wild-caught individuals from West Papua, Indonesia. Sequences were analysed from USA, the island of New Guinea and northern Australia, where the pet trade was thought to originate from. The size of the circles indicate the number of individuals identified with a given haplotype. Colours within the circles indicate the geographic provenance. Haplotypes that occurred in multiple locations are indicated with a pie chart showing the proportion of individuals from different locations. Single base pair substitutions are indicated as hatched black lines.

We took a subset of 140 individuals with complete mitochondrial data from USA and from the suspected founder populations in PNG, Indonesia and northern Australia to test for the origin of the pet species using network analysis. We observed a total of 142 variable sites forming 51 haplotypes (Hd = 0.9344; nucleotide diversity Pi = 0.03828). Tajima’s D was negative, −1.2, which can be indicative of a rapid population expansion, however this result was not significant (*P* = 0.10). We observed relatively low genetic distance values between Indonesia and USA populations (*F*_ST_ = 0.198; D_XY_ = 0.038; D_A_ = 0.008) (Table 1), which could reflect either the recent introduction history and/or ongoing gene flow. The median joining network shows 23 closely related USA haplotypes, two of which contain sequences identical to haplotypes from Sorong, Indonesia (H21 and H48) (Fig. 2). The USA and Australian populations are distantly related based on genetic divergence (*F*_ST_ = 0.86; D_XY_ = 0.097; D_A_ = 0.084). There is very little diversity within the nuclear network analysis of any population (Fig. S1). Only four haplotypes were observed in the total dataset, the most frequent occurred in all geographic locations, and all differed by only one base pair from each other. The three minor haplotypes occurred in Sol River, Indonesia (G-H1); Northern Territory, Australia (G-H2) and Texas, USA (G-H3).

**Table 1:** Gene flow and genetic differentiation statistics for *Petaurus breviceps* populations. Population statistics are presented as comparisons between (United States of America (USA), Indonesia (Indo), Papua New Guinea (PNG) and northern Australian (NAus), using F_*ST*_ (population differentiation), D_*xy*_ (absolute divergence) and D_*a*_ (net diversity).

**Statistic**	**Population**	**Indo**	**USA**	**NAus**
**F**_**st**_	**PNG**	0.242	0.599	0.572
	**Indo**	–	**0.198**	0.608
	**USA**	–	–	0.862
**D**_**xy**_	**PNG**	0.078	0.082	0.097
	**Indo**	–	**0.038**	0.101
	**USA**	–	–	0.097
**D**_**a**_	**PNG**	0.019	0.049	0.055
	**Indo**	–	**0.008**	0.061
	**USA**	–	–	0.084

**Figure 3:**
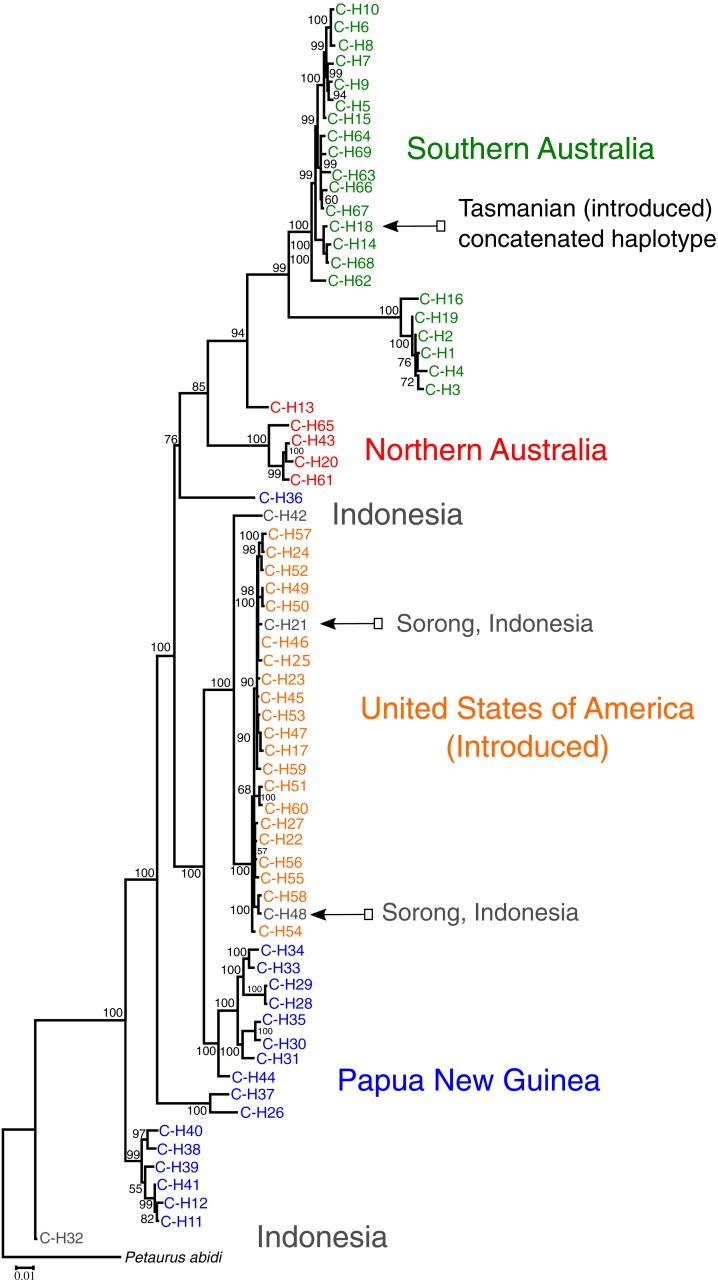
Maximum likelihood analysis demonstrating that *Petaurus breviceps* samples collected from the pet trade in the United States of America are most closely related to wild-caught individuals from Sorong, West Papua, Indonesia. Sequences from across the entire native and introduced range were included in the analysis. Evolutionary history was inferred by means of the maximum likelihood method using a concatenated dataset comprising mtDNA (ND2, ND4) and nDNA genes (*ω*-globin). The introduced population in USA (orange) falls directly within the Papuan clades (blue and grey). No Australian sequences (green and red) were identified within the USA population sampled. Bootstrap values are indicated on branch nodes.

Of the 287 individuals sequenced across the native range and the USA, 231 had complete data for all three genes and were concatenated for phylogenetic analysis. Maximum likelihood analysis shows the USA sequences are most closely related to sequences from Indonesia as expected (Fig. 3). The USA sequences fall within the PNG/Indonesian clades sharing a common ancestor with C-H42 (Kai Besar Island, Indonesia) (Malekian et al., 2010) and including two haplotypes, C-H21 and C-H48, which share identical sequence with Indonesian individuals sourced from Sorong, Indonesia (Campbell et al., 2018).

We used the intensely sampled Texas population to investigate within population genetic diversity. From the 106 samples with complete mitochondrial data we observed 75 variable sites forming 45 haplotypes (Hd = 0.9716; nucleotide diversity Pi = 0.00447). Tajima’s D was negative, −1.75, but not significant (*P* = 0.10). This result suggests a steady population expansion in Texas, however, it is difficult to rule out possible founder effects with such a short temporal scale.

A comparison of mitochondrial haplotype diversity between native and introduced populations of sugar gliders shows low diversity when compared with intrapopulation diversities among each of the native PNG, Indonesian and mainland Australian populations sampled (Table 2). However, the USA population has a much higher diversity when compared with an introduced population found in Tasmania, Australia, even though the species has been in the USA for a much shorter time period. This would suggest a recent introduction from multiple source populations into the USA, although ongoing gene flow cannot be ruled out.

**Table 2:** Mitochondrial haplotype diversity of native populations and introduced populations of *Petaurus breviceps*. The proportion of haplotypes per individual (Haplotype diversity) is reported to account for differing sampling intensities across studies and locations.

	**Native or introduced**	**No. of individuals**	**No. of haplotypes**	**Haplotype diversity**
USA combined	Introduced	112	23	0.21
Texas only	Introduced	106	22	0.21
Tasmania (AUS)^*^	Introduced	27	1	0.04
Papua New Guinea	Native	27	20	0.74
Indonesia	Native	5	4	0.80
Australia mainland^*^	Native	71	34	0.48

**Notes.**

* Asterisk indicates data from Campbell et al. (2018).

## Discussion

Our analysis suggests that all of the USA sugar gliders sampled in this study have originated from a source population in the vicinity of Sorong, Indonesia because all USA individuals exist in a single clade with high sequence identity to two individuals sampled from this region. Our sampling shows no evidence consistent with USA sugar gliders originating from other parts of Indonesia, Papua New Guinea or Australia. This suggests that trade in sugar gliders is from a concentrated area and there are no wide-reaching impacts of the pet-trade across the broader native range of sugar gliders. Our results are consistent with reports from legal Indonesian wildlife trade channels, which designate Sorong as the base for collection of sugar gliders and states that exported animals are either captive bred, or captured from surrounding wild locations. To abide by Indonesian regulations, exporters must restrict exportation to quotas of individuals (for example 225 animals for the years 2010 and 2011) (Lyons & Natusch, 2012). There is concern amongst conservation groups and non-governmental organisations (NGOs) working in the area that Indonesian exportation rates far exceed these quotas (Lyons & Natusch, 2012). Additionally, there are concerns about how wildlife quotas are calculated in Indonesia and where breeding facilities obtain animals if none currently exist in captivity (Janssen & Chng, 2018). Our study has not surveyed a sufficient number of genetic loci to provide quantitative estimates of exportation rate, and we are thus unable to determine compliance with export quotas. Encouragingly, we did not discover any unequivocal evidence for illegal trade of sugar gliders. However, we note that participation in this study from operators in the USA was voluntary. Additionally, our study has surveyed only a small proportion of the USA population (seven states). It is possible that with more extensive sampling from the USA population, cases of illegal trade may become apparent. However, because our sampling was focused upon two large operators that supply the majority of the country, we suggest that export from West Papua, Indonesia, is for the most part legal.

Compared to other introduced sugar glider populations, domestically kept exotic pet sugar gliders in the USA are a relatively diverse group, displaying 23 mitochondrial haplotypes. Traditionally introduced species are thought to harbour low genetic diversity due to small founding populations and bottlenecks (Nei, Maruyama & Chakraborty, 1975; Dlugosch & Parker, 2008; Frankham, 2010; Campbell et al., 2018). However, high diversity has been shown in introduced populations founded by multiple introductions (Kolbe et al., 2004; Facon et al., 2008; Bock et al., 2015). The genetic diversity for the USA population of sugar gliders is consistent with reports from breeders suggesting intermittent importation of new individuals into breeding colonies. The high mtDNA haplotype diversity we observed in USA captive populations is likely to be a result of multiple collection localities near Sorong and/or randomly introducing new haplotypes from a genetically diverse source population in West Papua. The high mitochondrial diversity in the USA and the low measures of genetic distance between the USA and Indonesia (*F*_ST_ = 0.198; D_XY_ = 0.038; D_A_ = 0.008) could suggest gene flow between the two countries or multiple recent introductions of individuals which were wild caught from varying localities. For comparison, the population of sugar gliders recently introduced to Tasmania, through a single introduction of unknown numbers approximately 150 years ago, consists of just a single haplotype (Campbell et al., 2018). Further, *F*_ST_ between Tasmanian and mainland populations is very high (*F*_ST_ = 0.680) but between population divergence statistics are very low (absolute diversity, D_XY_ = 0.008; net divergence, D_A_ = 0.005). The most basal lineage of all sugar gliders is haplotype C-H32, which originates from the geographical centre of the island of New Guinea in West Papua, Indonesia (Malekian et al., 2010). There is a strong separation between the PNG/Indonesian clades and the Australian clades.

## Conclusion

Here we have provided evidence to support anecdotal reports from commercial websites offering the sale of sugar gliders that the source of the USA population of sugar gliders is West Papua, Indonesia. In our sampling, we found no evidence of illegal trade from other parts for the sugar glider native range but we cannot discount the possibility that animals are taken from other parts of Indonesia or Papua New Guinea and traded from Sorong. The scope of our inference is limited due to sparse sampling of West Papua, surrounding islands and the voluntary nature of tissue donation from USA pet owners. More extensive sampling of wild sugar gliders, animals bred in captive breeding facilities in West Papua and privately owned sugar gliders in the USA along with detailed pedigree information from breeders in the USA would be required to fully understand the importation history (Hogg et al., 2018). We established that despite being founded very recently (less than 30 years ago), the USA population is significantly more diverse than other introduced but older populations in Australia (Campbell et al., 2018). This implies multiple recent introductions and/or gene flow between Indonesia and the USA (Dawnay et al., 2008; Ogden & Linacre, 2015), and suggests that there is sufficient diversity within the USA population to avoid negative consequences of inbreeding if pedigrees are carefully managed. Ongoing importation of sugar gliders from legal avenues would allow US breeders to actively manage genetic diversity in the captive USA population, while allowing Indonesian wildlife traders the opportunity to benefit from their natural resources. For the specific purpose of preventing inbreeding in the USA an appropriately managed wild harvest of sugar gliders from West Papua, Indonesia, could continue with sufficient regulation of wildlife trade and if sustainable wildlife harvest quotas are enforced (Nijman, 2010; Janssen & Chng, 2018). Initiatives to achieve this could include, stricter licensing and registration for exporters, minimum mandatory reporting standards for captive breeding facilities and monitoring of selected wildlife trade hubs (Nijman, 2010).

##  Supplemental Information

10.7717/peerj.6180/supp-1 Figure S1 Median joining network for sugar gliders in USA, Indonesia and Northern Territory, Australia, using the nuclear gene *ω* -globin

10.7717/peerj.6180/supp-2 Table S1 Exotic pet trade websites for sugar gliders in the USA Sugar glider websites containing anecdotal and sometimes conflicting information on the origin of the introduction, time of introduction and regularity of the introduction of sugar gliders into the USA.

10.7717/peerj.6180/supp-3 Table S2 Metadata for all individuals included in this study New samples collected for this study are indicated with an asterix (*). Adapted from (Campbell et al., 2018; Malekian, Cooper & Carthew, 2010; Malekian, Cooper & Carthew, 2010) . (ABTC: South Australia Museum, (A)M: Australia Museum, QM (Queensland Museum), M: Australian National Wildlife Collection CSIRO, U: Museum and Art Gallery of Northern Territory (MAGNT), A: Tasmanian Museum and Art Gallery (TMAG), QVM: Queen Victoria Museum and Art Gallery (QVMAG), AA/UC: University of Canberra Tissue Collection). Dash indicates missing data.

10.7717/peerj.6180/supp-4 Table S3 Nucleotide substitution models selected for data partitioning using the Akaike Information Criterion in MEGA v7.0

10.7717/peerj.6180/supp-5 Supplemental Information 1 Alignment file for Fig. 1

10.7717/peerj.6180/supp-6 Supplemental Information 2 Alignment file for Fig. 3

10.7717/peerj.6180/supp-7 Supplemental Information 3 DNA sequences for nuclear globin gene DNA sequences for the nuclear globin gene of sugar gliders collected in the USA.

10.7717/peerj.6180/supp-8 Supplemental Information 4 DNA sequences for the mitochondrial ND4 gene DNA sequences for the mitochondrial ND4 gene of sugar gliders collected in the USA.

10.7717/peerj.6180/supp-9 Supplemental Information 5 DNA sequences for the mitochondrial ND2 gene DNA sequences for the mitochondrial ND2 gene of sugar gliders collected in the USA.

## Additional Information and Declarations

### Data Availability

The following information was supplied regarding data availability: All genetic data in this study is available at Genbank, accession numbers MH247781–MH247925 and MH310446–MH310731. Table S2 lists the accession numbers for previously published samples and samples collected by this study. Sequence alignment files (NEXUS) for the figures are available as Supplementary Information.

## References

[ref-1] Alacs E, Georges A (2008). Wildlife across our borders: a review of the illegal trade in Australia. Australian Journal of Forensic Sciences.

[ref-2] Arevalo E, Davis SK, Sites JW (1994). Mitochondrial DNA sequence divergence and phylogenetic relationships among eight chromosome races of the *Sceloporus grammicus* complex (Phrynosomatidae) in central Mexico. Systematic Biology.

[ref-3] Bandelt HJ, Forster P, Röhl A (1999). Median-joining networks for inferring intraspecific phylogenies. Molecular Biology and Evolution.

[ref-4] Benson DA, Cavanaugh M, Clark K, Karsch-Mizrachi I, Lipman DJ, Ostell J, Sayers EW (2013). GenBank. Nucleic Acids Research.

[ref-5] Bock DG, Caseys C, Cousens RD, Hahn MA, Heredia SM, Hubner S, Turner KG, Whitney KD, Rieseberg LH, Hübner S, Turner KG, Whitney KD, Rieseberg LH, Harris J (2015). What we still don’t know about invasion genetics. Molecular Ecology.

[ref-6] Brust DM (2009). Sugar gliders: a complete veterinary care guide.

[ref-7] Bulte EH, Damania R (2005). An economic assessment of wildlife farming and conservation. Conservation Biology.

[ref-8] Bush ER, Baker SE, Macdonald DW (2014). Global trade in exotic pets 2006–2012. Conservation Biology.

[ref-9] Campbell CD, Sarre SD, Stojanovic D, Gruber B, Medlock K, Harris S, MacDonald AJ, Holleley CE (2018). When is a native species invasive? Incursion of a predatory marsupial detected using molecular and historical data. Diversity and Distributions.

[ref-10] Dawnay N, Ogden R, Thorpe RS, Pope LC, Dawson DA, McEwing R (2008). A forensic STR profiling system for the Eurasian badger: a framework for developing profiling systems for wildlife species. Forensic Science International: Genetics.

[ref-11] Dlugosch KM, Parker IM (2008). Founding events in species invasions: genetic variation, adaptive evolution, and the role of multiple introductions. Molecular Ecology.

[ref-12] Edgar RC (2004). MUSCLE: multiple sequence alignment with high accuracy and high throughput. Nucleic Acids Research.

[ref-13] Facon B, Pointier JP, Jarne P, Sarda V, David P (2008). High genetic variance in life-history strategies within invasive populations by way of multiple introductions. Current Biology.

[ref-14] Frankham R (2010). Challenges and opportunities of genetic approaches to biological conservation. Biological Conservation.

[ref-15] Hogg CJ, Dennison S, Frankham GJ, Hinds M, Johnson RN (2018). Stopping the spin cycle: genetics and bio-banking as a tool for addressing the laundering of illegally caught wildlife as “captive-bred”. Conservation Genetics Resources.

[ref-16] Janssen J, Chng SCLL (2018). Biological parameters used in setting captive-breeding quotas for Indonesia’s breeding facilities. Conservation Biology.

[ref-17] Johnson-Delaney CA (2006). Common procedures in hedgehogs, prairie dogs, exotic rodents, and companion marsupials. Veterinary Clinics of North America—Exotic Animal Practice.

[ref-18] Kearse M, Moir R, Wilson A, Stones-Havas S, Cheung M, Sturrock S, Buxton S, Cooper A, Markowitz S, Duran C, Thierer T, Ashton B, Meintjes P, Drummond A (2012). Geneious basic: an integrated and extendable desktop software platform for the organization and analysis of sequence data. Bioinformatics.

[ref-19] Koch AJ, Munks SA, Woehler EJ (2008). Hollow-using vertebrate fauna of Tasmania: distribution, hollow requirements and conservation status. Australian Journal of Zoology.

[ref-20] Kolbe JJ, Glor RE, Rodríguez Schettino L, Lara AC, Larson A, Losos JB (2004). Genetic variation increases during biological invasion by a Cuban lizard. Nature.

[ref-21] Kumar S, Stecher G, Tamura K (2016). MEGA7: molecular evolutionary genetics analysis version 7.0 for bigger datasets. Molecular Biology and Evolution.

[ref-22] Librado P, Rozas J (2009). DnaSP v5: a software for comprehensive analysis of DNA polymorphism data. Bioinformatics.

[ref-23] Lyons JA, Natusch DJD (2011). Wildlife laundering through breeding farms: illegal harvest, population declines and a means of regulating the trade of green pythons (*Morelia viridis*) from Indonesia. Biological Conservation.

[ref-24] Lyons JA, Natusch DJD (2012). Over-stepping the Quota? The trade in sugar gliders in West Papua, Indonesia. TRAFFIC Bulletin.

[ref-25] Malekian M, Cooper SJB, Carthew SM (2010). Phylogeography of the Australian sugar glider (*Petaurus breviceps*): evidence for a new divergent lineage in eastern Australia. Australian Journal of Zoology.

[ref-26] Malekian M, Cooper SJB, Norman JA, Christidis L, Carthew SM (2010). Molecular systematics and evolutionary origins of the genus *Petaurus* (Marsupialia: Petauridae) in Australia and New Guinea. Molecular Phylogenetics and Evolution.

[ref-27] Mockrin MH, Bennett EL, Labruna DT (2005). Wildlife farming: a viable alternative to hunting in tropical forests?. Wildlife Conservation.

[ref-28] Nei M, Maruyama T, Chakraborty R (1975). The bottleneck effect and genetic variability in populations. Evolution.

[ref-29] Nekaris KAI, Shepherd CR, Starr CR, Nijman V (2010). Exploring cultural drivers for wildlife trade via an ethnoprimatological approach: a case study of slender and slow lorises (*Loris* and *Nycticebus*) in South and Southeast Asia. American Journal of Primatology.

[ref-30] Nijman V (2010). An overview of international wildlife trade from Southeast Asia. Biodiversity and Conservation.

[ref-31] Ogden R, Linacre A (2015). Wildlife forensic science: a review of genetic geographic origin assignment. Forensic Science International: Genetics.

[ref-32] Osborne MJ, Christidis L (2001). Molecular phylogenetics of Australo-Papuan possums and gliders (family Petauridae). Molecular Phylogenetics and Evolution.

[ref-33] Smith M (1973). Petaurus breviceps. Mammalian Species.

[ref-34] Smith AP (1982). Diet and feeding strategies of the marsupial sugar glider in temperate Australia. Journal of Animal Ecology.

[ref-35] Stojanovic D, Webb MH, Alderman R, Porfirio LL, Heinsohn R (2014). Discovery of a novel predator reveals extreme but highly variable mortality for an endangered migratory bird. Diversity and Distributions.

[ref-36] Suckling G (1984). Population ecology of the sugar glider, *Petaurus breviceps*, in a system of fragmented habitats. Australian Wildlife Research.

[ref-37] Sukumaran J, Holder MT (2010). DendroPy: a python library for phylogenetic computing. Bioinformatics.

[ref-38] Sukumaran J, Holder MT (2015). https://github.com/jeetsukumaran/DendroPy.

[ref-39] Tajima F (1989). Statistical method for testing the neutral mutation hypothesis by DNA polymorphism. Genetics.

[ref-40] Wheeler D, Hope R, Cooper SB, Dolman G, Webb GC, Bottema CD, Gooley AA, Goodman M, Holland RA (2001). An orphaned mammalian beta-globin gene of ancient evolutionary origin. Proceedings of the National Academy of Sciences of the United States of America.

[ref-41] World Economic Forum (2017). Wildlife crime: a $23 billion trade that’s destroying our planet. https://www.weforum.org/agenda/2016/09/fighting-illegal-wildlife-and-forest-trade/.

[ref-42] Wyler LS, Sheikh PA (2008). International illegal trade in wildlife: threats and US policy. CRS report for congress.

[ref-43] Zwickl DJ (2006). Genetic algorithm approaches for the phylogenetic analysis of large biological sequence datasets under the maximum likelihood criterion. PhD dissertation.

